# Facial blindsight

**DOI:** 10.3389/fnhum.2015.00522

**Published:** 2015-09-29

**Authors:** Marco Solcà, Adrian G. Guggisberg, Armin Schnider, Béatrice Leemann

**Affiliations:** Division of Neurorehabilitation, Department of Clinical Neurosciences, University Hospital and University of GenevaGeneva, Switzerland

**Keywords:** blindsight, consciousness, face, anton’s syndrome, self recognition

## Abstract

Blindsight denotes unconscious residual visual capacities in the context of an inability to consciously recollect or identify visual information. It has been described for color and shape discrimination, movement or facial emotion recognition. The present study investigates a patient suffering from cortical blindness whilst maintaining select residual abilities in face detection. Our patient presented the capacity to distinguish between jumbled/normal faces, known/unknown faces or famous people’s categories although he failed to explicitly recognize or describe them. Conversely, performance was at chance level when asked to categorize non-facial stimuli. Our results provide clinical evidence for the notion that some aspects of facial processing can occur without perceptual awareness, possibly using direct tracts from the thalamus to associative visual cortex, bypassing the primary visual cortex.

## Introduction

Patients with damage to the geniculocalcarine visual pathways may present cortical blindness. Some of them demonstrate the capacity to correctly “guess” visual characteristics during forced choice task, reflecting unconscious visual perception. This residual visual ability without perceptual awareness is termed “blindsight” (Sanders et al., 1974; Weiskrantz et al., 1974; Stoerig and Cowey, 2007; Cowey, 2010) Visual pathways flowing directly from the retina to the superior colliculus (by passing the lateral geniculate nucleus (LGN)) or projections from the LGN to areas beyond striate cortex have been suggested to mediate blindsight behavior (Lamme, 2006; Stoerig and Cowey, 1997; Goebel et al., 2001; Tong, 2003; Stoerig and Cowey, 2007).

Various residual visual skills have been reported such as, detection of direction of movement (Sahraie et al., 1998), discrimination of simple shapes (Weiskrantz, 1986) or colors (Brent et al., 1994). More recently, cases of “affective blindsight” have been described. These patients have maintained the ability to discriminate between different facial emotions (de Gelder et al., 1999; Hamm et al., 2003).

Unconscious processing of other facial features have been described in several studies. For instance, patients who fail to recognize any face (i.e., prosopagnosia) may have different skin conductance in response to known vs. unknown faces (Bauer, 1984; Tranel and Damasio, 1985). Similarly, studies in healthy subject using visual masking to prevent stimulus awareness have revealed nonconscious recognition of facial identity at the behavioral (Stone and Valentine, 2004) and electrophysiological level (Henson et al., 2008).

Here we describe the first patient with cortical blindness presenting some advanced facial processing abilities, beyond simple processing of facial emotions.

## Materials and Methods

### Ethics Statement

The study was approved by the institutional ethical committees of Geneva and conducted according to principles outlined by the Declaration of Helsinki. The patient received an explanation of the research protocol and gave written informed consent.

### Patient Report

**Figure 1:**
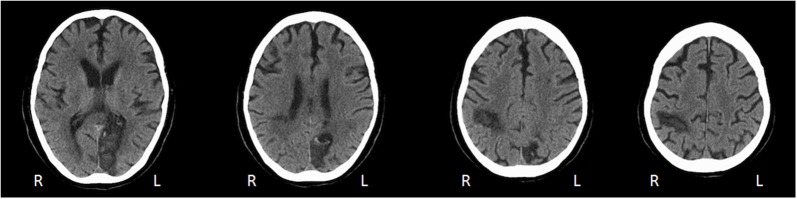
**Axial CT scan performed 6 weeks after stroke reveals right parieto-occipital and left occipital lesions**.

A.M. was a 67 years old right-handed man, with a medical history of cardiac arrhythmia that had required pacemaker implantation. He was admitted to the hospital for an elective Cryo-Maze-Procedure to treat new atrial fibrillation. The surgical intervention was complicated by perioperative ischemic stroke of the right parieto-occipital and of the left temporo-occipital cortices with secondary bilateral hemorrhagic transformation (Figure 1).

Clinically, A.M. presented Anton’s syndrome (Anton, 1899): he was totally blind but denied any visual deficit. He failed to detect a hand, hand movements, or a light source in any part of his visual field. When asked about what he saw, he described an imaginary environment, integrating elements perceived by other sensory modalities. For instance, he described a person that he heard and imagined that the person wore red clothes and a green hat. Over the following weeks, visual impairment improved slightly but remained extremely severe. At the time of the present investigation, between weeks 8 and 10 after the stroke, A.M. was still essentially blind and would only detect a strong light source in the left hemifield when the flashlight was directly pointing in his eyes; he would not detect it in other parts of the visual field or when it was not directly pointing in his eyes. However, he remained clinically blind (visual acuity < 20/200 tested with the Snellen Eye Chart) and failed to name, recognize or detect any object, color or movement. For example, when we waved the hand just in front of his eyes, he didn’t detect any change in the scene that he pretended to see and kept staring into the far space. A formal test of the visual field was not possible as the patient could not hold his gaze in one direction and the presence of Anton’s syndrome led to unreliable reports from the patient.

A surprising dissociation was observed between A.M’s virtually complete absence of vision and his behavior in certain life situations. For instance, he apparently was able to maintain eye contact with his examiners, and seemed to be familiar with some of his therapists on sight alone even though he could not name them. To assess this clinical impression we performed the following behavioral visual tests.

### Visual Assessment

We tested visual functions using forced choice tasks in which A.M. was instructed to identify the category of each image by choosing between two or four options (see below for details). Each test consisted of 20–24 images on white paper presented in random order for five seconds in the central visual field. Portrait images had approximately 17° horizontal and 22° vertical visual angle and square images 20° visual angle. Each task was repeated three times at different days resulting in a total of 60–72 items per task. Each test contained an equal number of each category leading to a chance level of 50% (two options task) or 25% (four options task).

**Figure 2:**
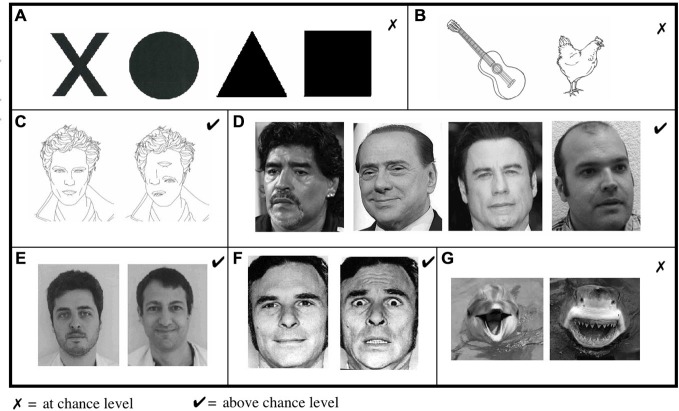
Visual assessment Showed impaired abilities in **(A)** shape and **(B)** object discrimination. **(C)** In contrast, A.M. was able to distinguish between jumbled and normal faces. **(D)** He was also able to distinguish between photographs of sportsmen, politicians, famous actors or unknown people and **(E)** to differentiate “known/unknown” faces of therapists from his and other medical departments. **(F)** He was also able to discriminate between fearful and neutral faces but **(G)** not when emotions were presented in other contexts.

*Shape perception* was assessed with a task in which A.M. had to guess the correct shape between black circles, squares, triangles, or crosses (Figure 2A). In a second experiment, he had to choose the drawing corresponding to a named category (animal or object; Figure 2B).

Forassessment of *face perception*, A.M. had first to recognize true faces in a set of jumbled or normal faces (Figure 2C). In a second task, we presented photographs of famous actors, politicians, sportsmen or unknown people and asked the patient to guess which of these four categories each item belonged to (Figure 2D). Then, he had to discriminate between known and unknown faces when presented with photographs of his therapists and therapists from other medical department (Figure 2E). In a complementary experiment, we presented A.M. with pictures of his own family and himself among unknown people.

For assessment of **emotion perception**, we presented photographs of fearful and neutral faces (Figure 2F; Ekman and Friesen, 1976) and tested his ability to recognize the correct emotion. In further sets, A.M. had to discriminate between fear provoking and non-fear provoking pictures of shark/dolphins (Figure 2G).

### Statistical Analysis

Discriminability between stimuli was calculated using signal detection theory (SDT; Macmillan and Creelman, 2005).

Statistical validity of the patient’s performance for each test was established using a chi-square goodness-of-fit test and tested against the null hypothesis of a performance equal to chance levels. Analyses were performed with R (R Development Core Team, 2013) including the “psyphy” package (Knoblauch, 2014).

## Results

A.M. failed to distinguish shape (Figure 2A; *d*′ = −0.3, 17% correct answers, chance level 25%, $\chi_{\left(1\right)}^{2}$ = 2.22, *p* = 0.13) and drawings of objects and animals (Figure 2B; *d*′ = 0.4, 58% correct answers, chance level 50%, $\chi_{\left(1\right)}^{2}$ = 1.67, *p* = 0.19).

In contrast, A.M. demonstrated remarkable residual capacity in face perception. He correctly distinguished true faces from jumbled faces (Figure 2C; *d*′ = 4.3, 82% correct answers, chance level 50%, $\chi_{\left(1\right)}^{2}$ = 24.07, *p* < 0.0001), and correctly guessed people’s category (Figure 2D; *d*′ = 1.7, 75% correct answers, chance level 25%, $\chi_{\left(1\right)}^{2}$ = 80, *p* < 0.0001). Similarly, A.M. was able to discriminate between his and other therapists (Figure 2E; *d*′ = 2.9, 93% correct answers, chance level 50%, $\chi_{\left(1\right)}^{2}$ = 49.47, *p* < 0.0001) and to distinguish fearful and neutral faces, (Figure 2F) with high accuracy (*d*′ = 4.9, 91%, chance level 50%, $\chi_{\left(1\right)}^{2}$ = 47.08, *p* < 0.0001). Conversely, his performance at detecting the emotional content of pictures/photographs without human faces (Figure 2G) was at chance level (*d*′ = 0.2, 53%: correct answers, chance level 50%, $\chi_{\left(1\right)}^{2}$ = 0.27, *p* = 0.6). In conclusion, scores in facial categorization tasks were significantly better than other tasks ($\chi_{\left(1\right)}^{2}$ = 77.56, *p* < 0.0001), although A.M. was unable to name or describe any of these faces and often misjudged their gender.

When presented with photographs of his own family he had strong feelings of familiarity although he failed to recognize them. For example, when looking at a photograph of his wife, he said: “he’s my best friend, he comes and visits me every day”. When presented with his own picture, he said: “It’s strange, I know him really well, I meet him every day but I cannot tell you who he is”.

## Discussion

**Figure 3:**
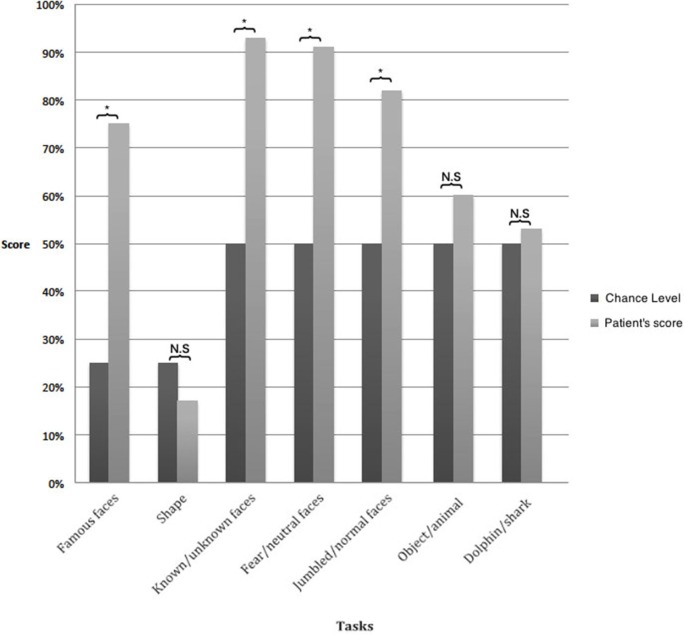
**Results of visual assessment.** The results of all tasks involving faces were significantly above chance whereas those without faces were at chance level. *p < 0.05.

Patient A.M. suffered from lesions of the left primary visual cortex and the right optic tract leading to cortical blindness, except for a strong light source in the left hemifield. He failed to discriminate shapes and objects, thus showing no evidence of blindsight as it has been traditionally described (Cowey, 2010). Despite his apparent blindness, he maintained some remarkable capacities for facial processing (Figure 3). He distinguished between drawings of a correctly composed and a jumbled face beyond chance. While he failed to recognize the identity of any face, he was able to categorize a significant proportion of faces as known or unknown, according to category, and regarding the facial expression. We propose to call this disorder “facial blindsight” as the patient often correctly guessed the category of faces without any explicit knowledge about their identity. As evident from Figure 2, these specific capacities cannot be attributed to differences in the visual contrast of recognized vs. non-recognized items. On the contrary, while faces generally have a lower contrast when compared to basic polygons, A.M. was still more capable of recognizing them.

Blindsight patients are usually unaware of the visual stimulus. A.M. was unaware of the stimuli that were truly shown to him although he would confabulate stimuli that were not present in reality. His facial recognition abilities thus appear to be due to unconscious processes whose result he could not explicitly report or verbalize. For instance, when looking at his wife’s picture, he pretended to recognize his best friend. We assume that this confabulation was driven by some “feeling of knowing” but also illustrates the absence of the perception of concrete facial features.

Despite the fact that cases of Anton syndrome associated with blindsight have been previously reported (Maddula et al., 2009; Carota et al., 2013), alternative diagnosis explaining A.M. clinical features could be considered. Some patients with severe visual agnosia have been reported to have relatively spared abilities in facial perception whereas shape perception was impaired (Moscovitch et al., 1997; McMullen et al., 2000; Germine et al., 2011). However, these patients had intact visual abilities on a basic sensory level and could for instance detect hand movements or count fingers (Moscovitch et al., 1997; McMullen et al., 2000; Germine et al., 2011). As A.M. was clinically blind, he did not fulfill the basic criteria of agnosia.

Even if other clinical condition cannot be formally excluded given some limitations of the present case (concomitant Anton syndrome and impossibility to perform visual field), clinical and experimental evidence clearly supports the presence of a blindsight phenomenon. The neural circuit responsible for blindsight seems to involve a subcortical colliculo-pulvinar pathway independent of the primary visual cortex (Schmid et al., 2010). Observations in patients with affective blindsight (Morris et al., 2001; Williams et al., 2004; Pegna et al., 2005) have shown amygdala activation through a V1-independent pathway. Our patient not only presented the ability to distinguish facial emotions but also more general skills in face perception, suggesting a more complete involvement of the neural system for face perception, including mainly the lateral fusiform gyrus, the superior temporal sulcus and the inferior occipital Gyri (Haxby et al., 2000; Schmidt et al., 2005).

In our patient, the right temporo-occipital junction with the fusiform gyrus was intact. This area is critical for face perception. Prosopagnosia, the inability to recognize the identity of faces, is always due to right-sided or bilateral lesions of this area. Imaging studies on the recognition of facial identity and general face pattern consistently showed activation of the lateral fusiform gyrus (Haxby et al., 2000; Hoffman and Haxby, 2000) with right hemisphere dominance (Kanwisher et al., 1997; McCarthy et al., 1997; Halgren et al., 1999; Rossion et al., 2000). Moreover, studies in healthy subject have demonstrated that faces can elicit specific activity in the right lateral fusiform gyrus even when they are not consciously perceived (Morris et al., 2007; Kouider et al., 2009). Hence, we surmise that A.M. unconscious residual visual faculties are due to activation of the right lateral fusiform gyrus through direct tracts from thalamic nuclei bypassing the primary visual cortex.

While various types of blindsight have been described, A.M. is, to our knowledge, the first case of facial blindsight. This case provides evidence that some aspects of face processing can occur unconsciously and independently of the primary visual cortex.

## Conflict of Interest Statement

The authors declare that the research was conducted in the absence of any commercial or financial relationships that could be construed as a potential conflict of interest.
